# Occupational exposure of platinum-based anti-cancer drugs: five-year monitoring of hair and environmental samples in a single hospital

**DOI:** 10.1186/s12995-020-00280-1

**Published:** 2020-09-29

**Authors:** Ai Hori, Mari Shimura, Yutaka Iida, Kazuhiko Yamada, Kyoko Nohara, Takayuki Ichinose, Ai Yamashita, Junko Shirataki, Shotaro Hagiwara

**Affiliations:** 1 Department of Epidemiology and Prevention, National Center for Global Health and Medicine (NCGM), Tokyo, Japan; 2 Department of Global Public Health, Faculty of Medicine, University of Tsukuba, 1-1-1, Tennodai, Tsukuba, Ibaraki 305-8577 Japan; 3 Department of Intractable Diseases, NCGM, 1-21-1, Toyama, Shinjuku-ku, Tokyo, 162-8655 Japan; 4 Inorganic Analysis Laboratories, Toray Research Center, Inc., Otsu, Japan; 5 Department of Surgery, Hospital, NCGM, Tokyo, Japan; 6 Division of Hematology, Internal Medicine, Hospital, NCGM, Tokyo, Japan; 7 Present address: Department of Hematology, School of Medicine, Tokyo Women’s Medical University, Tokyo, Japan

**Keywords:** Platinum, Anti-cancer drug, Hospital, Hair, Inductively coupled plasma mass spectrometry

## Abstract

**Background:**

Occupational exposure to chemotherapeutic agents in hospitals is a critical issue. Here, we focused on occupational exposure to platinum-based anti-cancer drugs (PDs) by evaluating platinum concentrations in hair and environmental workplace samples to monitor the risk among workers.

**Methods:**

Hospital workers who dealt with or without PDs, patients treated with PDs, and non-medical office workers outside the hospital donated hair samples and completed a questionnaire regarding their history of handling PDs, including any incidents. Hair samples were collected and surface wipe sampling was performed in July 2010 and April 2015, before and after moving to a new building and introducing a revised safety program in August 2010. Samples were analyzed by inductively coupled plasma-mass spectrometry.

**Results:**

Platinum concentrations in hair from PDs-handling workers was significantly higher than in non-PDs-handling workers (*P* = 0.045), although 50 times lower than that from PDs-treated patients. Platinum concentrations in the hospital environment had decreased at the second survey 5 years later but had not changed significantly in the hair samples from hospital workers.

**Conclusion:**

Platinum concentrations in hair are likely dependent on the frequency of handling PDs. Reduced environmental contamination from PDs did not influence platinum levels in hospital workers’ hair. Continuous monitoring by measuring platinum concentrations in the environment and in hair would provide information regarding these issues.

## Introduction

Anticancer drugs are therapeutic but are also occupational hazards for hospital workers. The handling of anticancer drugs is known to be associated with adverse reproductive outcomes [1], such as spontaneous abortion [2, 3], premature delivery [2], and low birth weight [4] among female hospital workers. Cisplatin and other platinum-containing anticancer drugs that are commonly used in hospital settings are classed as group 2A carcinogens, or as group 1 carcinogens in combination with etoposide and bleomycin, according to the International Agency for Research on Cancer [5].

Environmental contamination with platinum-based drugs (PDs) originating in hospital wards [6] and pharmacies [7, 8] has been well documented. PDs have been detected in hospital workers’ body fluids such as urine and blood [9, 10]. Studies suggest that PDs could contaminate the hospital environment through vaporization when intravenous solutions are being prepared for cancer patients and result in exposure via the skin and respiratory system. Therefore, governmental and academic guidelines recommend minimizing the exposure of hospital workers to anticancer drugs through the proper use of safety cabinets, closed-system drug transfer devices, and personal protection equipment (PPE) [11, 12].

Sampling of urine and blood from volunteers is commonly performed. However, hair sampling would be less invasive and would allow for easy storage and long survival of samples even at room temperature. Hair samples have been taken previously to monitor exposure to heavy metals such as cadmium [13], lead [14], and mercury [15] as a result of exposure to industrial waste. Hair samples from cancer patients undergoing PD treatment have also been taken to evaluate the PDs level in patients [16, 17]. These findings suggest that hair would be also useful to quantify the PDs level in hospital workers. However, the monitoring of exposure to PDs from hair samples of hospital workers is not well documented.

In this study, we used ICP-MS technologies to evaluate exposure to trace-level occupational PDs by measuring hair and workplace environmental samples among hospital workers and comparing them with samples from patients and non-hospital office workers.

## Methods

### Sample donors

Doctors, nurses, and pharmacists were recruited from five hospital departments—three oncology wards (hematology, respiratory, and gastroenterology), a diabetes ward, and the pharmacy—at the Center Hospital of the National Center for Global Health and Medicine (NCGM), Tokyo. Hair samples were collected and environmental surface wipes were performed at the hospital in July 2010 in an old building (40 years old) and again in April 2015 in a new building (5 years old); that is, before and after a move to a new building in August 2010 and accompanying revision of the safety management program for hazardous drugs, based on the regulations of the Ministry of Health, Labour and Welfare of Japan. The participants were 13 men and 46 women (age 22–49 years; response rate 74%) in 2010 and 24 men and 52 women (age 23–60 years, response rate 84%) in 2015. Two hospital workers were able to participate in both surveys. All participants completed a questionnaire asking about personal information (sex, age, division, and occupation); occupational handling of PDs, including incidents; and use of personal protective equipment (PPE) such as caps, glasses, masks, gowns, gloves, and foot covers.

For comparative analysis, 15 patients with esophageal cancer at the hospital donated hair samples in 2015, who were receiving platinum-based drugs (PDs). Fifteen non-medical office workers (*n* = 15) working outside of the hospital also donated hair samples in 2015. All participants provided written informed consent via research collaborators or their medical doctors who contributed as co-authors. An explanation of the study was provided including hair sampling which would impose a minimal burden, and patients would be informed of their analytical value if they requested it. Chief doctors of each department were informed of the study protocol and asked to approve the collection of participant questionnaires and hair samples. This study was approved by the Ethics Committee of NCGM (#NCGM-G-00623-02, #NCGM-G-000845-02, #NCGM-G-001766-00), in accordance with the Declaration of Helsinki of the World Medical Association. All participants provided written informed consent.

### Measurement of platinum from hair samples

Participants were asked to pluck hair samples including the follicles. Each single hair sample was then weighed to within a 0.01 mg accuracy and digested in HNO_3_ and H_2_O_2_ with heating. After cooling and dissolving the digest with 4% vol aqua regia up to 4 mL, iridium was added as an internal standard for analysis. Platinum concentrations were analyzed using inductively coupled plasma sector field mass spectrometry (ICP-SFMS; Element XR, Thermo-Fisher Scientific, MA). The lower limit of platinum quantification was estimated as 10 times the standard deviation of signals from continuous measurements of 10 blank samples, according to Japanese Industrial Standards K0133 [18, 19]. The limit of quantification (LOQ) was 0.001411 ng/mL in 2010 and 0.001271 ng/mL in 2015, suggesting that the measurements in both years had similar sensitivity and specificity. Line scans of hairs were analyzed by LA-ICP-MS (New Wave Research UP213, ESI Japan, Tokyo, Japan and ELAN DRC II, PerkinElmer, MA). Each hair was set with adhesive tape and pre-ablation was performed to remove a surface part of the hair with the following settings: beam spot size, 55 μm; 0.06 J/cm^2^; scan speed, 20–80 μm/s. Ablation was then used to analyze ^194^Pt and ^195^Pt: beam spot size, 40 μm; 1–2 J/cm^2^; scan speed, 20–80 μm/s.

### Measurement of platinum from environmental surface wipe samples

Surface wipe samples were collected from the hair sample donors’ offices in the three oncology wards, the pharmacy, and in the diabetes ward (control ward) at the general hospital and from the non-medical office outside the hospital. The diabetes ward was used as a control; however, a few patients who received PD therapy were admitted to the ward due to their clinical condition or due to bed management. We used filter paper cut in half and moistened with 70% ethanol (Ultrapure ethanol, Wako, Tokyo, Japan) to sample 20 cm × 20 cm patches of the environmental surfaces. Every location was sampled twice, with a 2-week interval in an independent occasion. Each sample was placed in HNO_3_ and H_2_O_2_ and digested by heating. After cooling the solution was digested with 4% vol aqua regia up to 10 mL, and iridium was added as an internal standard for the analysis. Platinum concentration was quantified using inductively coupled plasma quadrupole mass spectrometry (ICP-QMS; ELAN DRC II, PerkinElmer). The LOQ was 0.007116 ng/mL in 2010 and 0.009379 ng/mL in 2015, suggesting that the data from both years were comparable.

### Statistical analysis

Medians and interquartile ranges of platinum weight (picogram [pg]) and platinum concentrations per single hair sample (nanogram [ng/g]) were calculated according to the participant’s characteristics and compared using the Kruskal-Wallis test. Platinum concentrations from the hospital environment in 2010 and 2015 are presented as median and interquartile range (pg/cm^2^) and were compared using the Wilcoxon rank-sum test. Statistical tests were two-sided and regarded as statistically significant at *P* < 0.05. Analysis was performed using Stata SE version 14.0 (College Station, TX, USA) and GraphPad Prism, version 7.0.

## Results

### Platinum contaminations in hair determined by line scanning

**Fig. 1:**
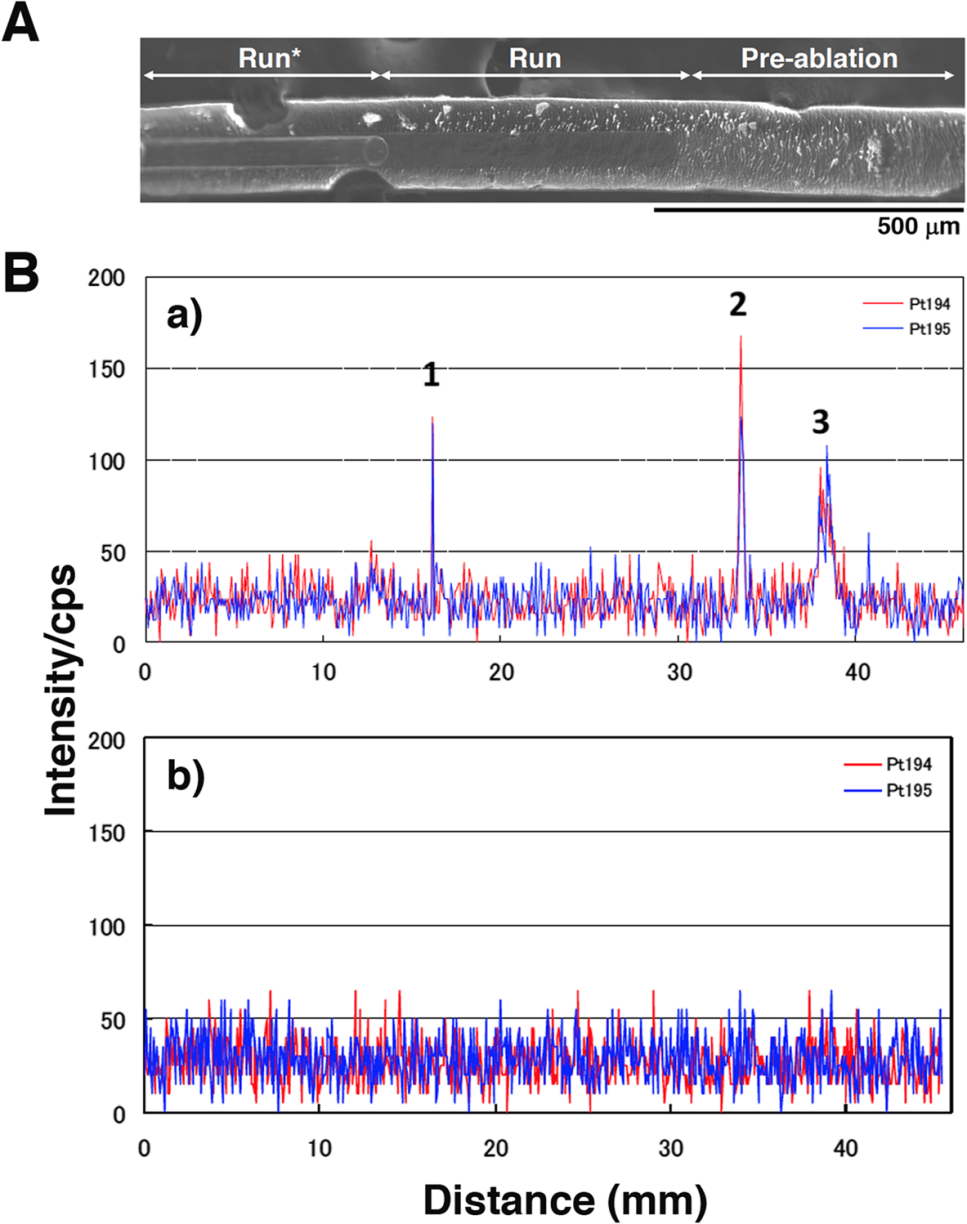
Line scanning of a representative hair sample from hospital workers. **A**. SEM image of a hair by laser ablation. Pre-ablation was performed before the measurements. Run, measurements of platinum in this study; Run*, second measurement. **B**. **a)** Three indentical peaks in the hair (**1, 2, 3**) were obtained for both ^194^Pt and ^195^Pt in one hospital worker. **b)** No identical platinum peaks detected in a hair sample from another hospital worker

**Fig. 2:**
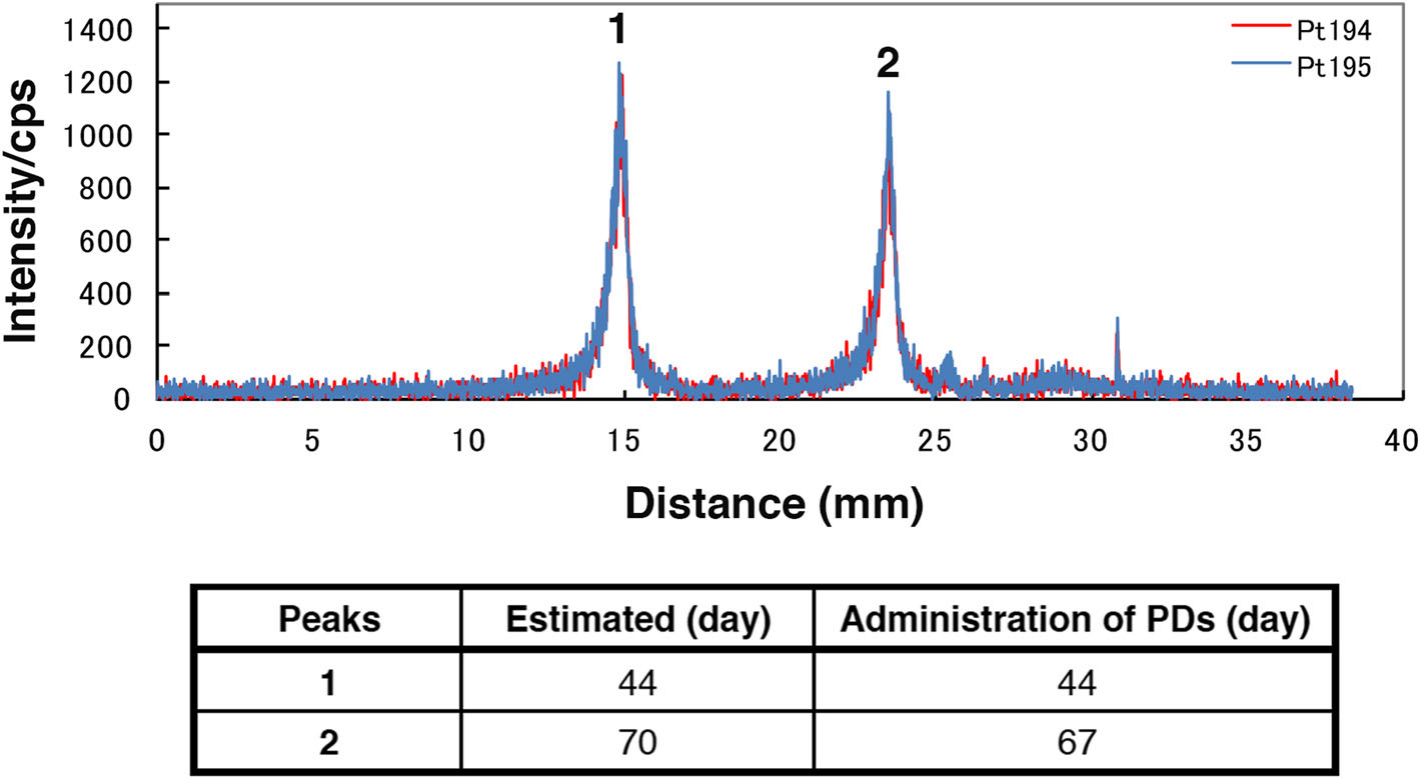
Line scanning of a representative hair sample from patients treated with PDs. Two indentical peaks in the hair (**1, 2**) were obtained for both ^194^Pt and ^195^Pt in this patient treated with PDs. Peaks reflect the administration of PDs. Estimated: days before the hair sampling that were estimated by measuring the distance from the follicle to platinum peaks, based on an average growth rate of 10 mm/month. Administration of PDs: days before the hair samplings that were noted in clinical records of PD administration

Line scanning of hair by LA-ICP-MS reveals past uptake of PDs from the root to the tip, with hair generally growing 0.3–0.4 mm/day (Fig. 1a). Figure 1Ba is a representative sample from a hospital worker collected before the move to the new building and the introduction of the revised safety management program for hazardous drugs. We found three clear positive platinum peaks at both ^194^Pt and ^195^Pt. Of particular note, the shape of the third peak is similar to that seen in patients treated with PDs (Fig. 2), although the peak counts were lower. The time between the first and second peaks was estimated at 1.7 months and that between the second and third peaks at 0.4 months (Fig. 1Ba). Although we could not determine the exact date when that particular hospital worker handled PDs, PDs were often handled while preparing carboplatin solution on the oncology ward late at night, due to the pharmacy being closed. The hospital worker used neither proper personal protective equipment (PPE) nor a safety cabinet. On the other hand, another worker at the hospital had no remarkable platinum peak above background in the hair sample (Fig. 1Bb), suggesting that the peaks in Fig. 1Ba are positive signals for platinum. These data indicate that platinum peaks can be detected in hair samples not only from patients receiving PDs but also from hospital workers. We also could not exclude the possibility that the hospital worker showing platinum peaks had absorbed PDs into the body. These findings led us to hypothesize that workers handling PDs might be at risk from exposure and prompted us to conduct further investigation at the hospital over the next 5-year period.

### Platinum concentrations in hair

Table 1 shows the characteristics of the participants of the surveys conducted in 2010 and 2015.

**Table 1:** Description of study participants

Ward/ Items		2010	2015	***P***-value^d^
**Diabetes**	**Total number**	10	19	
	**Gender, Women**^**a**^	9 (90)	12 (63)	0.201
	**Age, Median (interquartile range)**	28 (26–35)	29 (26–33)	0.610
	Doctor	0 (0)	5 (26)	0.134
	Nurse	10 (100)	14 (74)	
	Pharmacist	0 (0)	0 (0)	
	**PD Users**^**b**^	3 (30)	8 (42)	0.694
**Hematology**	**Total number**	20	16	
	**Gender, Women**	14 (70)	14 (88)	0.257
	**Age, Median (interquartile range)**	29 (26–33)	29 (24–38)	0.711
	Doctor	5 (25)	2 (12)	0.426
	Nurse	15 (75)	14 (88)	
	Pharmacist	0 (0)	0 (0)	
	**PD Users**	13 (65)	13 (81)	0.456
**Respiratory**	**Total number**	11	13	
	**Gender, Women**	10 (91)	9 (69)	0.327
	**Age, Median (interquartile range)**	26 (25–34)	27 (25–30)	0.786
	Doctor	0 (0)	5 (38)	0.041
	Nurse	11 (100)	8 (62)	
	Pharmacist	0 (0)	0 (0)	
	**PD Users**	9 (82)	7 (54)	0.211
**Gastro-enterology**	**Total number**	8	13	
	**Gender, Women**	6 (75)	12 (92)	0.531
	**Age, Median (interquartile range)**	28 (27–31)	28 (23–30)	0.680
	Doctor	2 (25)	3 (23)	1.000
	Nurse	6 (75)	10 (77)	
	Pharmacist	0 (0)	0 (0)	
	**PD Users**	6 (75)	10 (77)	1.000
**Pharmacy**	**Total number**	10	15	
	**Gender, Women**	7 (70)	5 (33)	0.111
	**Age, Median (interquartile range)**	31 (29–35)	33 (28–43)	0.692
	Doctor	0 (0)	0 (0)	–
	Nurse	0 (0)	0 (0)	
	Pharmacist	10 (100)	15 (100)	
	**PD Users**	5 (50)	13 (87)	0.075
**Total hospital workers**	**Total number**	59	76	
	**Gender, Women**	46 (78)	52 (68)	0.247
	**Age, Median (interquartile range)**	29 (26–33)	29 (26–36)	0.803
	Doctor	7 (12)	15 (20)	0.378
	Nurse	42 (71)	46 (60)	
	Pharmacist	10 (17)	15 (20)	
	**PD Users**	36 (61)	51 (67)	0.475
**Patients**^**c**^	**Total number**	–	15	
	**Gender, Women**		1 (7)	–
	**Age, Median (interquartile range)**		64 (57–70)	–
**Non-medical office workers**	**Total number**	–	15	
	**Gender, Women**		10 (67)	–
	**Age, Median (interquartile range)**		41 (37–47)	–

^a^ Values are number (percentage) unless otherwise stated

^b^ Platinum-containing drug (PDs) handling within the past 3 months answered in questionnaire

^c^ Treated with PDs

^d^ Fisher’s exact test for categorical variables and Wilcoxon rank-sum test for age

**Fig. 3:**
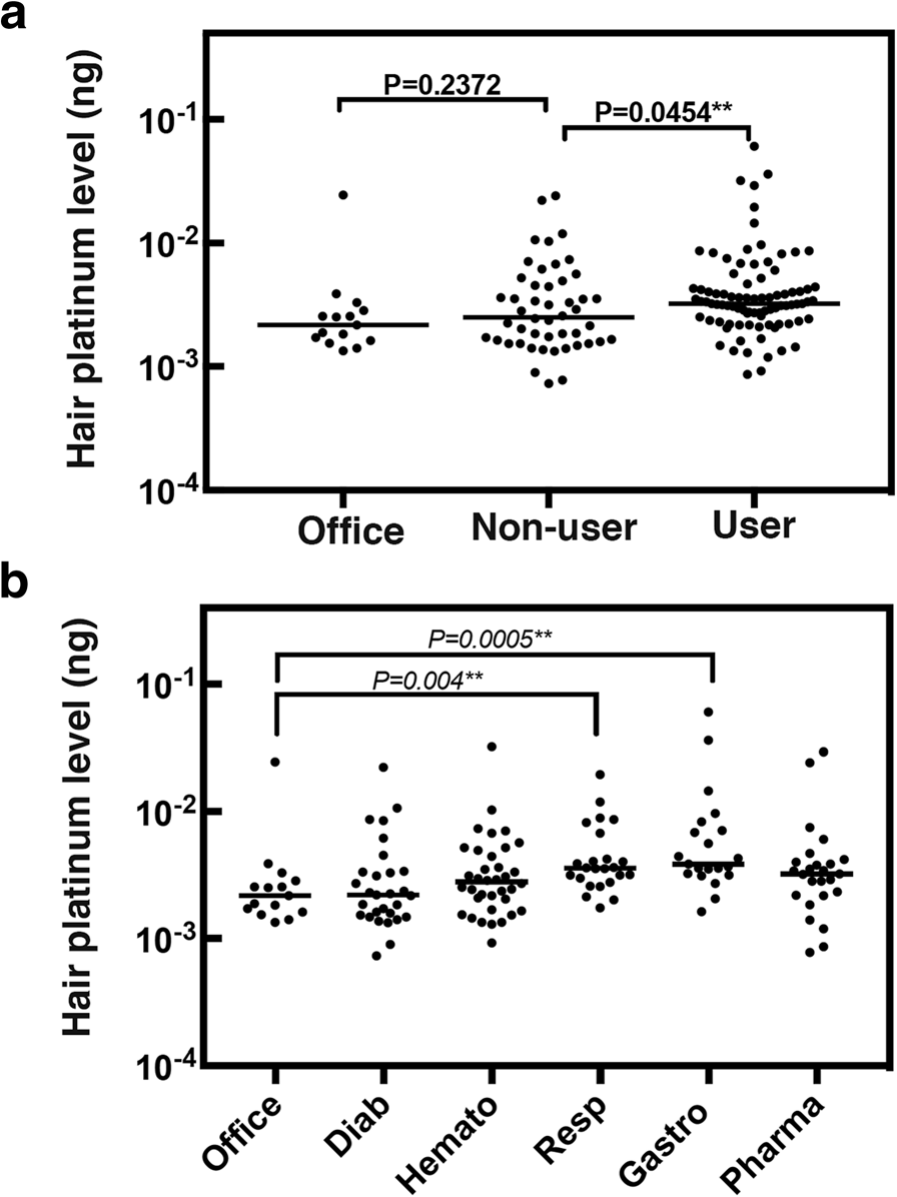
Hair platinum concentration among hospital workers using data from both 2010 and 2015. **a** Hair platinum concentration in all hospital workers. **b** Hair platinum concentration in hospital workers according to department. Office, non-medical office staff not handling PDs; Non-users, hospital workers not handling PDs within the previous 3 months. User; hospital workers handling PDs within the previous 3 months. Diab, diabetes; Hemato, hematology; Resp, respiratory; Gastro, gastroentorology; Pharmac, pharmacy; ng, nanogram. Values indicate the results of Kruskal-Wallis tests. **, *P* < 0.05

Age, sex, and occupations were not significantly different between 2010 and 2015 (Table 1). Notably, among the hospital workers, 61% in 2010 and 67% in 2015 had handled PDs within the previous 3 months (Table 1). Using data from both 2010 and 2015, platinum concentrations in hair from workers handling PDs (users) were significantly higher than those from workers not handling PDs (non-users) (*P* = 0.045, Fig. 3a and Table 2). Hairs from non-users contained levels similar to those from the non-medical office workers (Office, Fig. 3a and Table 2). Notably, the platinum levels in hair from users were more than 50 times lower than those from patients treated with PDs (Table 2).

**Table 2:** Hair platinum levels of participants

	Number	Hair platinum levels (× 10^−3^ ng^c^)			
		Median (interquartile range)	Min	Max	Variance
**Office workers**	15	2.17 (1.62–2.85)	1.34	24.56	33.78
**All hospital workers (2010–2015)**					
Users of PDs^a^	87	3.24 (2.35–4.42)	0.86	60.43	71.25
Non-users	48	2.51 (1.61–4.74)	0.73	24.24	22.86
**Patients**^b^	15	213.16 (31.90–627.25)	0	1230.46	150.214

^a^ Platinum-containing drug (PDs) handling within the past 3 months answered in questionnaire

^b^Patients treated by standard preoperative chemotherapy certified by medical record

^c^*ng* Nanogram

We next evaluated the platinum levels in hair from hospital workers according to department. Respiratory and gastroenterology staff had significantly higher levels than non-medical office workers (*P* = 0.004 and *P* = 0.0005, respectively; Fig. 3b). Workers handling PDs on both of these wards were higher than those on the diabetes ward (Table 1). Although hematology and pharmacy staff had similar percentages to staff handling PDs, their platinum levels in hair were not significantly different from those of non-medical office workers, suggesting good management of exposure to PDs in these departments (Fig. 3b).

### Platinum concentrations determined by environmental surface wipe sampling

**Fig. 4:**
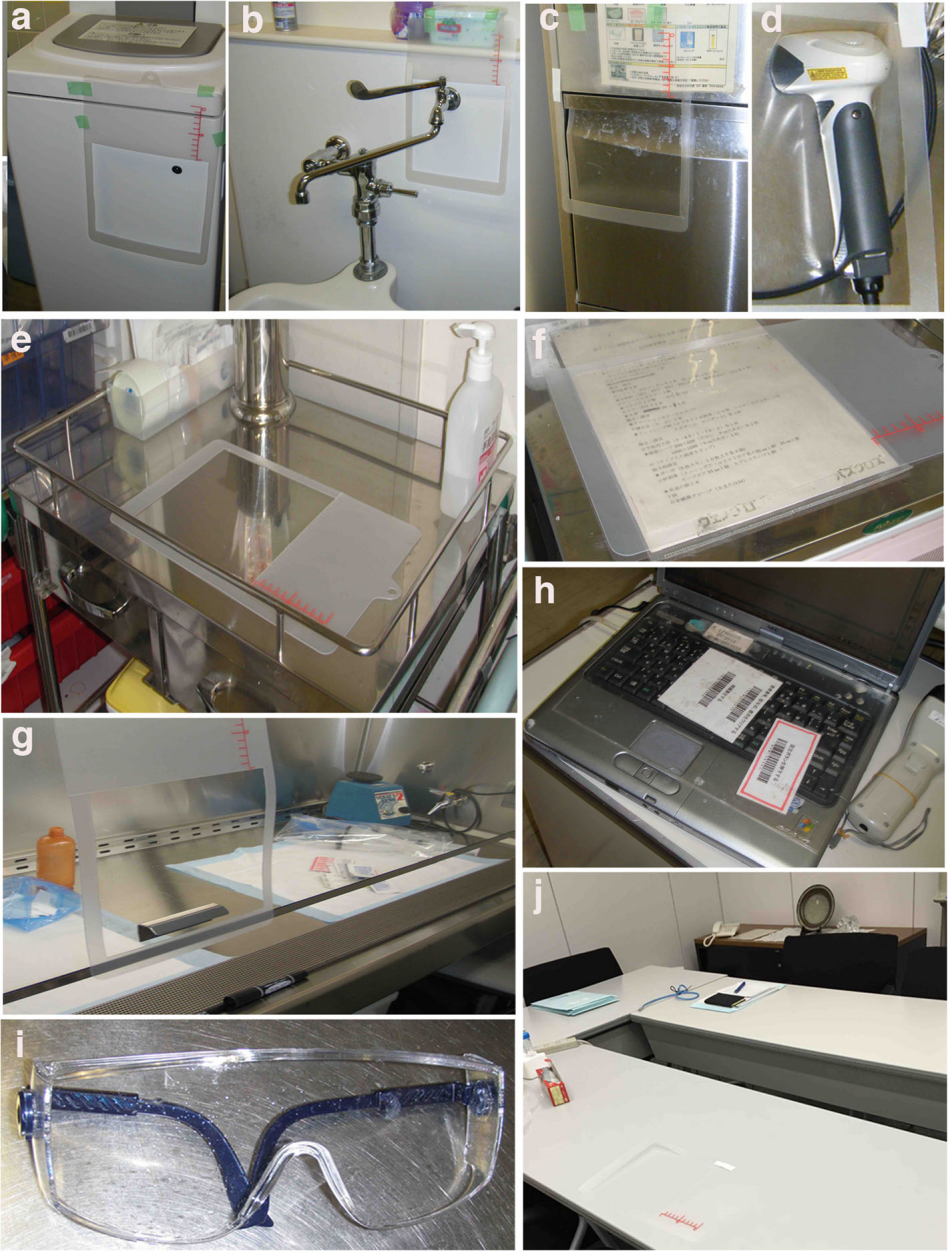
Photographs showing the surfaces in wipe sampling. **a** a device for measuring urine quantity, **b** sanitary room tap handle, **c** device for washing urine pots, **d** bar cord reader, **e** drug mixing table, **f** plastic film on drug mixing table, **g** front window of safety cabinet, **h** keyboard cover on computer, **i** protective glasses for drug mixing, and **j** office meeting table

Environmental platinum concentrations at sampling locations in the hospital are shown in Table 3 (see also photographs in Fig. 4). The highest platinum level in 2010 was found on a device for measuring urine in the sanitary room of the respiratory ward (1050 picograms [pg]/cm^2^), where nurses or caregivers disposed of urine from patients. In contrast, the highest levels in 2015 were found on a drug mixing table in the gastroenterology ward (3.75 pg/cm^2^) and a safety cabinet in the pharmacy (3.75 pg/cm^2^). These findings suggest that environmental contamination from handling urine samples had improved by 2015.

**Table 3:** Platinum levels in surface wipes before and after induction of hospital safety management system

2010 Sampling spot	Platinum levels* (pg/cm^2^)**		2015 Sampling spot	Platinum levels* (pg/cm^2^)**	
**Diabetes ward**			**Diabetes ward**		
Drug mixing table	N.D.	N.D.	Drug mixing table	N.D.	N.D.
Device for measuring urine quantity^a^	2.28	3.25	Device for washing urine pot	N.D.	0.45
Doorknob	N.D.	N.D.	Doorknob	N.D.	0.45
Meeting table	N.D.	N.D.	Sanitary room tap handle ^b^	N.D.	0.53
**Hematology ward**			**Hematology ward**		
Drug mixing table 1^e^	0.85	0.30	Drug mixing table 1	N.D.	N.D.
Drug mixing table 2	0.4	0.6	Drug mixing table 2	N.D.	N.D.
Drug mixing table 3	0.85	2.13	Meeting table	0.6	N.D.
Plastic film on drug mixing table 3 ^f^	10	3.25	Wagon for mixed hazardous drug	N.D.	N.D.
Device for measuring urine quantity	0.28	N.D.	Device for washing urine pot ^c^	N.D.	N.D.
Doorknob	N.D.	N.D.	Doorknob	N.D.	N.D.
Keyboard cover on computer ^h^	15.25	4.5	Sanitary room tap handle	N.D.	0.38
**Gastroenterology ward**			**Gastroenterology ward**		
Drug mixing table	3	1.75	Drug mixing table	0.63	**3.75**
Handle of drug mixing table	N.D.	0.93	Wagon for mixed hazardous drug	N.D.	N.D.
Device for measuring urine quantity	2.18	6.25	Device for washing urine pot	N.D.	N.D.
Doorknob	0.73	6.5	Doorknob	N.D.	N.D.
Meeting table	7	0.3	Sanitary room tap handle	N.D.	N.D.
**Respiratory ward**			**Respiratory ward**		
Drug mixing table 1	1.35	0.35	Drug mixing table	N.D.	1.6
Drug mixing table 2	0.55	0.3	Wagon for mixed hazardous drug	N.D.	N.D.
Device for measuring urine quantity	**1050**	157.5	Device for washing urine pot	N.D.	0.35
Doorknob	0.6	0.53	Doorknob	N.D.	N.D.
Protective glasses for drug mixing ^i^	2	1.08	Sanitary room tap handle	N.D.	0.25
**Pharmacy**			**Pharmacy**		
Inside safety cabinet	5.25	3.75	Inside safety cabinet	0.55	**3.75**
Front window of safety cabinet	3.5	2.33	Front window of safety cabinet ^g^	N.D.	N.D.
Doorknob	0.28	0.35	Doorknob	2.28	2.03
Wagon for mixed hazardous drug	3.75	9	Tray for mixed hazardous drug	N.D.	N.D.
Floor	1.65	1.73	Desk surface	0.48	N.D.
Chair	3.25	1.83	Bar cord reader ^d^	0.38	0.75
			**Non-medical office**		
			Office meeting table ^j^	0.3	N.D.
			Coffee table	N.D.	N.D.
			Office doorknob	0.8	0.28
			Washstand surface	N.D.	N.D.
			Mail service desk	N.D.	N.D.

N.D. not detected. For a-j, please see pictures in Fig. 4

*Every location was sampled twice, with a 2-week interval in an independent occasion

**pg, picogram

**Fig. 5:**
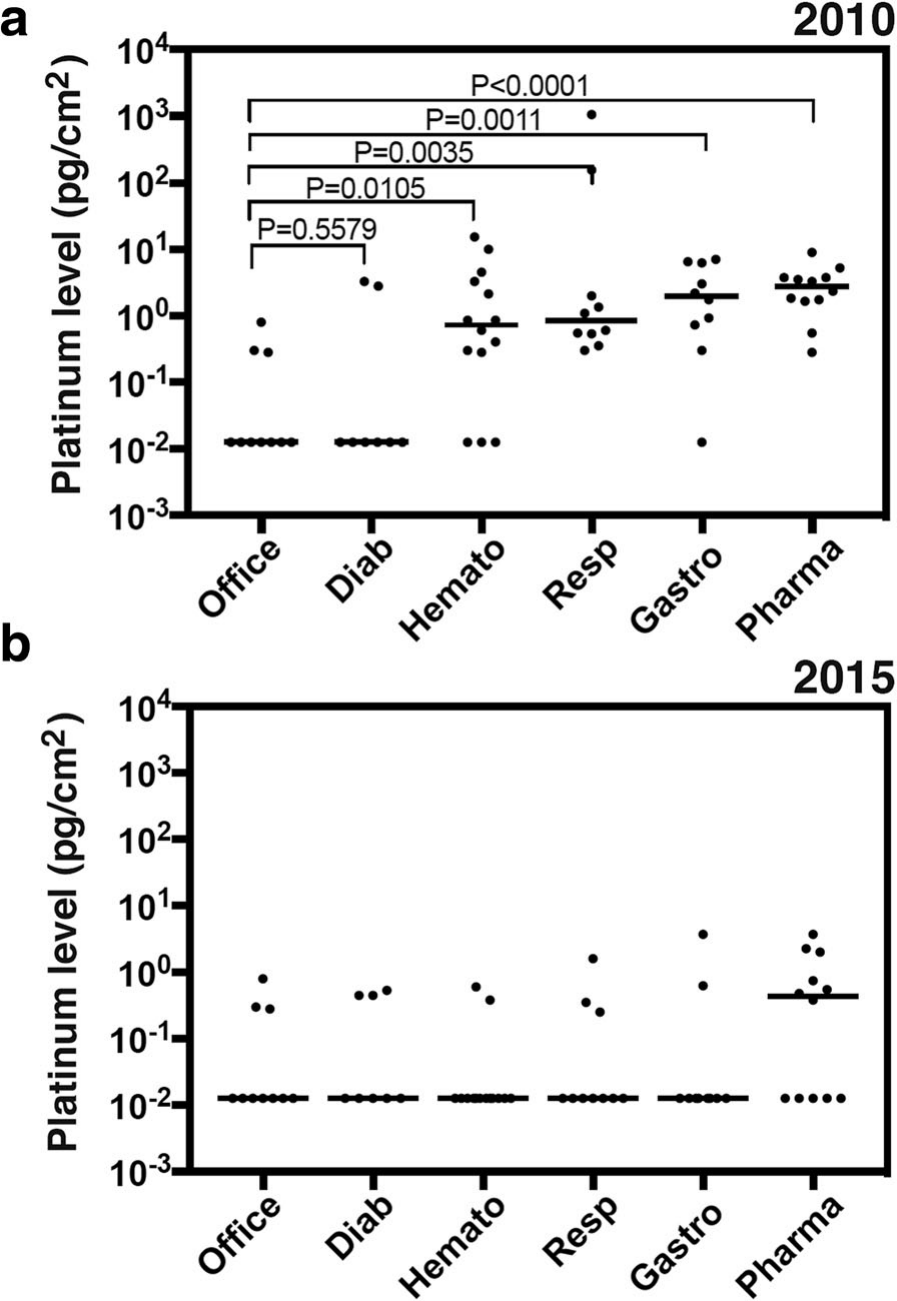
Environmental platinum levels at the hospital. Environmental platinum levels in each department in (**a**) 2010 and (**b**) 2015. Office, non-medical office staff not handling PDs; Diab, diabetes; Hemato, hematology; Resp, respiratory; Gastro, gastroenterology; Pharmac, pharmacy; pg, picogram. Values indicate Kruskal-Wallis testing with levels of significance (*P* < 0.05)

**Fig. 6:**
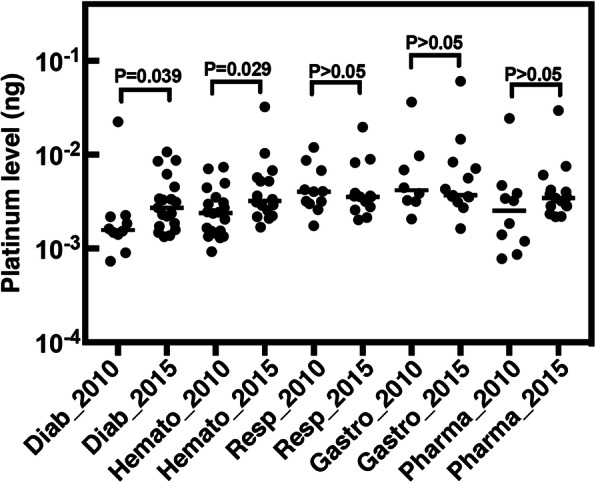
Hair platinum concentration levels in hospital workers according to department between 2010 and 2015. Diab, diabetes; Hemato, hematology; Resp, respiratory; Gastro, gastroenterology; Pharma, pharmacy; ng, nanogram. Values indicate Kruskal-Wallis test. **, *P* < 0.05

When the environmental platinum concentrations were compared for each department, they were found to be significantly higher on the oncology wards (except of Diabetes ward) than in the non-medical office environment outside the hospital in 2010 (Fig. 5a). Notably, two measurements showing relatively high platinum levels in the diabetes ward, as detected by a device for measuring urine quantity in 2010 (Fig. 5a and Table 3), could have resulted from a few patients who received PD therapy in the diabetes ward at that time, which suggests that evaluation depending on the ward is not always effective. In contrast, the concentrations in most departments was not significantly different from the non-medical office environment in 2015 (Fig. 5b). This suggests that environmental platinum concentrations in the hospital had decreased 5 years after the move to the new building and the accompanying introduction of the revised safety management program; however, it is notable that platinum concentrations in hair from hospital workers did not improve significantly (Fig. 6).

## Discussion

### Monitoring method using hair samples

Although there are several reports of platinum-positive results in blood and urine samples from hospital workers, few studies have assessed hair samples by ICP-MS for the monitoring of occupational exposure to PDs. Hair sampling has advantages over blood or urine testing, including its less invasive nature, the easy storage and long survival time of hair samples, and the ability to determine exposure history from the samples. Notably, our data from patients treated with PDs showed that the peak in hair samples was delayed compared with the peak in plasma (Additional file 1: Figure S1). This may be due to a time lag for accumulation in hair via the circulation, suggesting that platinum detection in hair was positively related to the platinum concentration in plasma. However, the assessment of trace-level occupational exposure to platinum through monitoring was at one time controversial [20]; it was not known whether platinum levels from biological samples were related to tracing exposure to PDs. Fundamentally, we could not rule out the possibility of platinum from sources other than PDs, such as dental appliances, metallic platinum dust, and catalysts in car exhaust systems [20]. In fact, our data showed that one person, who had the highest platinum level among office workers, may have been exposed to sources of platinum other than PDs. It is notable that the platinum levels of office workers were similar to those of PD non-users at the hospital. Additionally, platinum levels in PD users at the hospital were higher than those of PD non-users with statistical significance. The data suggest a high likelihood that the platinum detected in PD users was derived from PDs. Certain control groups are essential in order to estimate the source of platinum. A recently developed procedure that involves line scanning by LA-ICP-MS shows peaks with resolution adequate enough to identify the date of exposure to PDs [17]. In fact, the error between a date detected by LA-ICP-MS and the actual date of PDs treatment is within 3 days (Fig. 2). Errors can derive from individual hair growth rates. This technology has the advantage of being able to estimate the source of platinum based on dated events in hospital workers’ records. Our data suggest that hair sampling is a useful and reliable method for monitoring exposure to PDs.

### Decrease in environmental PDs contamination

Environmental contamination by PDs was significantly reduced in all oncology departments after the move to the new building and accompanying introduction of the revised safety management program. There are a number of possible reasons for this. First, since 2010 it has been prohibited to mix PDs anywhere but in a 24-h pharmacy, where the drugs are strictly regulated by using a ventilated safety cabinet and a closed-system transfer device [21, 22]; this is likely the major reason. Second, hospital workers now measure patients’ urine less often due to switching to a patient self-return scheme, which is consistent with the decreased values found in urine-related locations in 2015 (Table 3 and Fig. 4). Because urine contains relatively high concentrations of PDs after treatment [23], careful consideration must be taken in the handling of urine from patients treated with these drugs. When considering the recent increase in the number of outpatients receiving chemotherapy or home medical care, a safety manual is essential not only for hospital workers but also for patients and their families at home. Third, positive attitudes toward the revised safety management program [24] had improved PDs contamination in the pharmacy. Notably, in 2015, the platinum levels in the pharmacy tended to be lower despite increased handling of PDs.

### Hair platinum concentrations did not change despite change in the environment

Despite the improvement in environmental contamination by PDs, concentrations in hospital workers’ hair was not affected (Fig. 6). This lack of change in the hair samples may be because, first, we could not follow the same donor population from 2010 to 2015. Except for two workers, all others had left the hospital and were replaced by workers from other hospitals; in other words, new incoming workers showed exposure to PDs in their hair. Second, hair can contain past exposure. If hair is long enough (e.g., hair as long as 36 cm may keep past exposure up to 3 years earlier), ICP-MS measurement will show it. LA-ICP-MS, on the other hand, which can determine exposure history, may reveal changes in hair. Third, hospital workers were still experiencing incidents in the handling of PDs such as from needle stick injuries or spills (Table 4). Finally, there might have been PDs hot spots in areas other than those used for the wipe samplings. Continuous monitoring or follow-up of the same group by measuring platinum concentrations in the environment and in hair would provide information regarding these issues.

**Table 4:** Platinum based anti-cancer drug exposure within past three months

	2010			2015		
	No. of workers with experience			No. of workers with experience		
	Doctor	Nurse	Pharmacist	Doctor	Nurse	Pharmacist
	(*n* = 7)	(*n* = 42)	(*n* = 10)	(n = 15)	(*n* = 46)	(n = 15)
**Handling experience**						
Drug mixing	4	1	5	0	0	12
Bottle or line handling	2	28	0	1	37	0
**Incident cases**						
Needle stick injury	0	0	0	0	0	1
Spill when mixing drugs	0	1	2	0	0	2
Spill when bottle or line handling	0	5	0	0	0	0

### Limitations

Our study had several limitations. First, it was a single-center survey and, therefore, careful interpretation of the data is required. Second, the changes in environmental platinum levels observed before and after moving to the new building may not simply reflect a decrease in PDs contamination because we cannot rule out the possibility that there was an accumulation of PDs contamination in the old building despite daily cleaning and maintenance. Only continuous monitoring would resolve this limitation.

## Conclusion

This study suggested trace level contamination from exposure to PDs among hospital workers that likely depended on the frequency of handling PDs. A significant reduction has been seen in environmental platinum contamination in the 5 years after the revision of a safety management program and move to a new building; however, the environmental influence on levels of PDs in hospital workers’ hair was not clearly obtained in this study. Hair sampling is a useful and reliable method for monitoring the level of exposure to PDs among hospital workers. Continuous monitoring or follow-up of the same group by measuring platinum concentrations in the environment and in hair would provide further information.

## Supplementary information

**Additional file 1: Figure S1.** Platinum concentration levels in patients’ hair and plasma after treatment with PDs.

## Data Availability

The datasets used and/or analysed during the current study are available from the corresponding author on reasonable request.
