# Supplementary figures and images for: Occupational exposure of platinum-based anti-cancer drugs: five-year monitoring of hair and environmental samples in a single hospital
Source: J Occup Med Toxicol. 2020 Sep 29;15:29. doi: 10.1186/s12995-020-00280-1 (PMC7523399; doi:10.1186/s12995-020-00280-1)

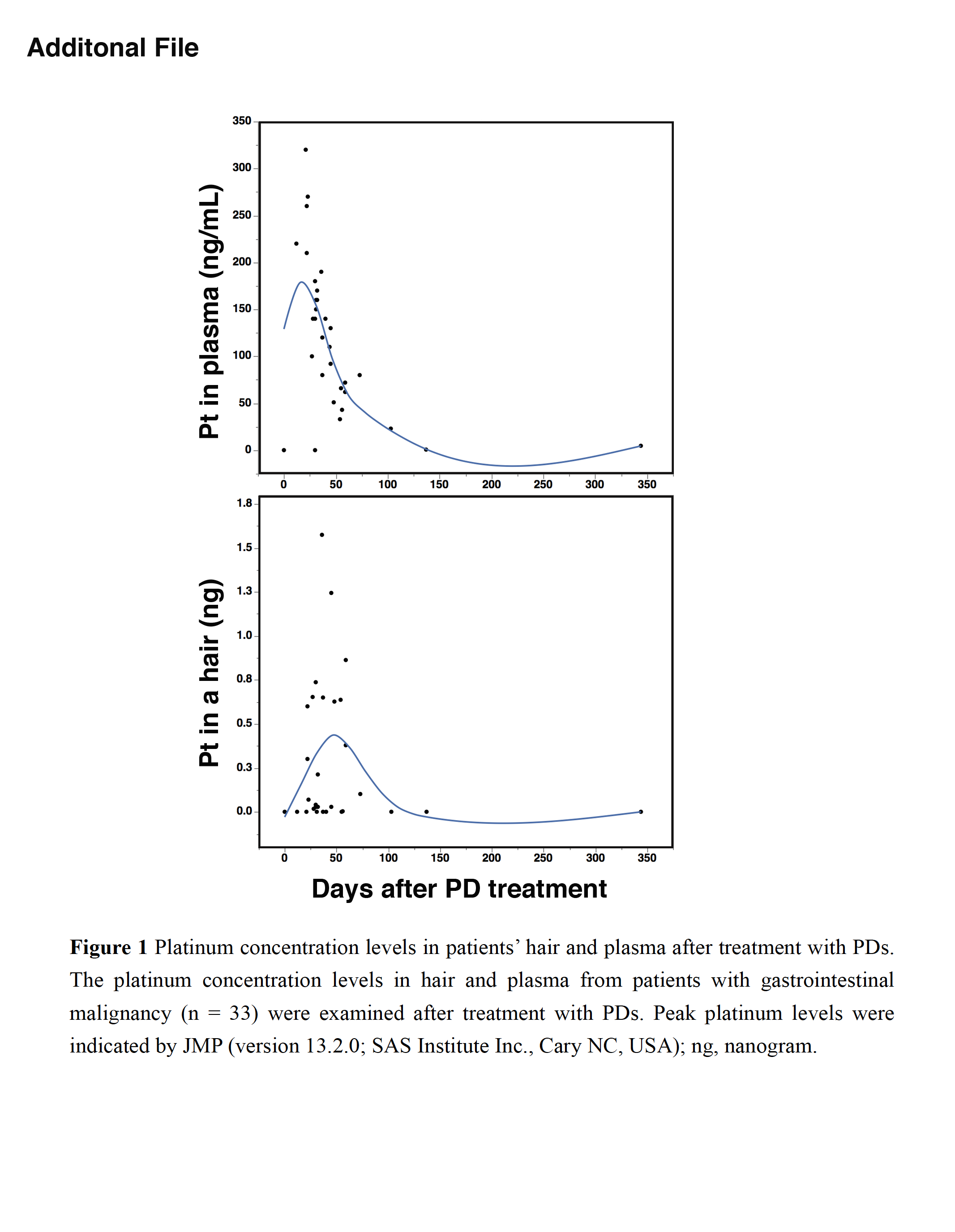

Supplement: Supplementary file 1 — Additional file 1: Figure S1. Platinum concentration levels in patients’ hair and plasma after treatment with PDs. [file 12995_2020_280_MOESM1_ESM.tif]
